# Retraction: *PDHA1* gene knockout in prostate cancer cells results in metabolic reprogramming towards greater glutamine dependence

**DOI:** 10.18632/oncotarget.28777

**Published:** 2025-11-06

**Authors:** Yaqing Li, Xiaoran Li, Xiaoli Li, Yali Zhong, Yasai Ji, Dandan Yu, Mingzhi Zhang, Jian-Guo Wen, Hongquan Zhang, Mariusz Adam Goscinski, Jahn M. Nesland, Zhenhe Suo

**Affiliations:** ^1^Department of Oncology, The First Affiliated Hospital of Zhengzhou University, Zhengzhou, Henan Province, China; ^2^Department of Pathology, The Norwegian Radium Hospital, Oslo University Hospital, University of Oslo, Montebello, Oslo, Norway; ^3^Department of Pathology, The Institute of Clinical Medicine, Faculty of Medicine, University of Oslo, Montebello, Oslo, Norway; ^4^Institute of Clinical Medicine, The First Affiliated Hospital of Zhengzhou University, Zhengzhou, Henan Province, China; ^5^Department of Anatomy, Histology and Embryology, Peking University Health Science Center, Peking University, Beijing, China; ^6^Department of Surgery, the Norwegian Radium Hospital, Oslo University Hospital, University of Oslo, Oslo, Norway

**This article has been retracted:** Oncotarget has completed its investigation of this paper. Oncotarget received an email from Frode Jahnsen, Head of Research Department of Pathology at Oslo University Hospital. His correspondence stated that the Commission on Research Integrity at Oslo University Hospital and Institute of Clinical Medicine, University of Oslo, had completed an investigation and concluded that there was evidence of scientific misconduct. More specifically, in Figure 4C the experiments and handling of data were intentionally performed in a manner in which the presentation of data is incorrect. The Commission found serious misinterpretations of data, concluding that they believe Figure 4C has been manipulated. As well, the Commission discovered that in Figure 4D, the histogram is not identical to the underlying background data and in Figure 5D, it is likely that the histogram was intentionally manipulated. They found that the underlying background data are missing for several of the Figures. The corresponding author of the paper, Zehnhe Suo, has reviewed all documentation, but has not been able to provide any new information to change the conclusion stated above.

We also found that two panels of bright field cells images in Figure 2D overlap with panels in Figure 5A, illustrating different experiments. The first author of the article, with the agreement of the corresponding author and on behalf of all the co-authors, confirmed the negligence at figure preparation and inability to resolve all the concerns, and asked for the withdrawal. In the light of the above facts, the editorial decision was made to retract the paper, but Oncotarget was not able to receive the signatures of the authors.

Original article: Oncotarget. 2016; 7:53837–53852. 53837-53852. https://doi.org/10.18632/oncotarget.10782

